# Intensive neurorehabilitation and allogeneic stem cells transplantation in canine degenerative myelopathy

**DOI:** 10.3389/fvets.2023.1192744

**Published:** 2023-07-13

**Authors:** Débora Gouveia, Jéssica Correia, Ana Cardoso, Carla Carvalho, Ana Catarina Oliveira, António Almeida, Óscar Gamboa, Lénio Ribeiro, Mariana Branquinho, Ana Sousa, Bruna Lopes, Patrícia Sousa, Alícia Moreira, André Coelho, Alexandra Rêma, Rui Alvites, António Ferreira, Ana Colette Maurício, Ângela Martins

**Affiliations:** ^1^Arrábida Veterinary Hospital, Arrábida Animal Rehabilitation Center, Setubal, Portugal; ^2^Superior School of Health, Protection and Animal Welfare, Polytechnic Institute of Lusophony, Lisboa, Portugal; ^3^Faculty of Veterinary Medicine, Lusófona University, Lisboa, Portugal; ^4^Faculty of Veterinary Medicine, University of Lisbon, Lisboa, Portugal; ^5^Departamento de Clínicas Veterinárias, Instituto de Ciências Biomédicas de Abel Salaza, Universidade do Porto, Porto, Portugal; ^6^Centro de Estudos de Ciência Animal, Instituto de Ciências, Tecnologias e Agroambiente da Universidade do Porto, Porto, Portugal; ^7^Associate Laboratory for Animal and Veterinary Science (AL4AnimalS), Lisboa, Portugal; ^8^Instituto Universitário de Ciências da Saúde (IUCS), Cooperativa de Ensino Superior Politécnico e Universitário (CESPU), Gandra, Portugal; ^9^CIISA - Centro Interdisciplinar-Investigáo em Saúde Animal, Faculdade de Medicina Veterinária, Av. Universi dade Técnica de Lisboa, Lisboa, Portugal

**Keywords:** mesenchymal stem cells, intensive neurorehabilitation, dogs, degenerative myelopathy, locomotor training, electrical stimulation

## Abstract

**Introduction:**

Degenerative myelopathy (DM) is a neurodegenerative spinal cord disease with upper motor neurons, with progressive and chronic clinical signs, similar to amyotrophic lateral sclerosis (ALS). DM has a complex etiology mainly associated with SOD1 gene mutation and its toxic role, with no specific treatment. Daily intensive rehabilitation showed survival time near 8 months but most animals are euthanized 6–12 months after clinical signs onset.

**Methods:**

This prospective controlled blinded cohort clinical study aims to evaluate the neural regeneration response ability of DM dogs subjected to an intensive neurorehabilitation protocol with mesenchymal stem cells (MSCs) transplantation. In total, 13 non-ambulatory (OFS 6 or 8) dogs with homozygous genotype DM/DM and diagnosed by exclusion were included. All were allocated to the intensive neurorehabilitation with MSCs protocol (INSCP) group (*n* = 8) or to the ambulatory rehabilitation protocol (ARP) group (*n* = 5), which differ in regard to training intensity, modalities frequency, and MSCs transplantation. The INSCP group was hospitalized for 1 month (T0 to T1), followed by MSCs transplantation (T1) and a second month (T2), whereas the ARP group was under ambulatory treatment for the same 2 months.

**Results:**

Survival mean time of total population was 375 days, with 438 days for the INSCP group and 274 for the ARP group, with a marked difference on the Kaplan–Meier survival analysis. When comparing the literature's results, there was also a clear difference in the one-sample *t*-test (*p* = 0.013) with an increase in time of approximately 70%. OFS classifications between groups at each time point were significantly different (*p* = 0.008) by the one-way ANOVA and the independent sample *t*-test.

**Discussion:**

This INSCP showed to be safe, feasible, and a possibility for a long progression of DM dogs with quality of life and functional improvement. This study should be continued.

## 1. Introduction

Degenerative myelopathy (DM) is a neurodegenerative spinal cord disease characterized by the progressive and chronic onset of clinical signs in large-breed dogs (1, 2). These signs are mainly of T3-L3 neuro-localization and include proprioceptive ataxia and spastic paraparesis (3, 4), similar to those presented in human patients with amyotrophic lateral sclerosis (ALS) (5).

Most dogs are between 5 and 14 years of age (6) and clinical signs are presented on average in 9 years old (3, 7), regardless of gender and other diseases (3, 8).

This disease has a prevalence of 0.19% in dogs (1, 7) but a specific prevalence of 2.01% in the German shepherd (1), which is the first described breed with DM (9). However, different authors have already described histological confirmation of the disease in other breeds, such as Siberian Husky (10), Miniature Poodle (11), Boxer (12–14), Pembroke Welsh Corgi (1, 15–17), Rhodesian ridgeback (12), Chesapeake Bay Retriever (12), Bernese Bouvier, Kerry Blue terrier, Golden Retriever, Pug (3) and also mixed breed dogs (9). There are also other described breeds but without histological confirmation (6).

Degenerative myelopathy has a complex and unknown etiology (7), but recent studies have been developed demonstrating the SOD1 gene mutation as one of the main causes (18). This gene presents as a homodimer that converts superoxide radicals into hydrogen and oxygen peroxide (5). Thus, superoxide dismutase (SOD), an enzyme involved in the dismutase of superoxide radicals, has three SOD isoforms: cytoplasmic (SOD1), mitochondrial (SOD2), and secreted outside the cell (SOD3). Also, some research in DM has highlighted two SOD1 mutant proteins (E40K and T18S) as insoluble and capable of inducing a toxic role associated with these isoforms (19–21).

There are several differential diagnoses related to geriatric dogs and T3-L3 spinal cord lesions (e.g., intervertebral disc disease (IVDD) Hansen type II, neoplasia, and meningoencephalomyelitis) (4). Therefore, *antemortem* diagnosis depends on the clinical history (the onset and progression of neurological signs), upper motor neurons (UMNs) clinical signs compatible with T3-L3 myelopathy, absence of spinal cord compressive lesion, and inflammatory changes on the cerebrospinal fluid (CSF). The gold standard complementary exam is magnetic resonance imaging (MRI) but computed tomography/CT is also performed to exclude disc disease, discospondylitis, and neoplasia (4, 22).

Thus, for the final diagnosis of DM, it is necessary to have compatible history and clinical signs (9), the presence of SOD1 gene mutation (5), and the exclusion of other spinal cord diseases (4, 7). Nevertheless, DM may coexist with other diseases of the nervous system, resulting in the need for an accurate diagnosis in the future, for example, through specific biomarkers (23). According to a study carried out in 2017, a suitable biomarker would be the neurofilament heavy chain (pNFH) that is released into the interstitial fluid during axonal injury and neurodegeneration (24).

The connection between DM and ALS is based on the genetic similarities of both diseases, that is, the mutation in the SOD1 nucleotide (25). In ALS, approximately 20% of cases have the SOD1 mutation (26) transmitted as an autosomal dominant disease, whereas DM is an autosomal recessive one (5). Furthermore, the results of studies performed in dogs with DM may allow a better understanding of the effectiveness of therapeutic interventions in the treatment of ALS (3).

There is no specific treatment for DM (6, 27). The medical pharmacological approach is based on non-steroidal anti-inflammatory drugs or steroids, such as prednisolone, due to its effect on the management of neurological signs, however with low evidence with regard to disease progression (28).

Other therapeutic approaches include an initial protocol based on active physical exercises and supplementation with Vitamin B, Vitamin E, Aminocaproic acid, and *N*-acetylcysteine (8, 29).

Aminocaproic acid blocks inflammation pathways and reduces fibrin degradation (29). On the other hand, *N*-acetylcysteine, a glutathione precursor, scavenges free radicals, preventing the activation of enzymes that cause tissue damage (30). Vitamin supplementation can contribute to the improvement of DM dogs, inhibiting the release of prostaglandins and cytokines that act on the inflammation cascade (29).

Degenerative myelopathy dogs that are not on a multimodal approach reach end-stage disease within 6 months of initial diagnosis (29, 31). A study by Kathmann et al. (6) reported that daily physiotherapy increased survival time in these dogs. In 22 DM dogs, the ones that had intensive physiotherapy protocol showed survival of nearly 8 months, when compared to a moderate protocol (~4 months) or without any protocol (~2 months). In another study with dogs under a similar protocol, 15–20% did not show worsening of neurological status, with some dogs surviving for more than 4 years (31). Thus, the prognosis of DM remains to be poor and, in most cases, dogs are euthanized between 6 and 12 months after the first clinical signs (1).

Regenerative medicine is the branch of medicine that promotes tissue regeneration and consequent functional recovery through the regrowth or replacement of injured cells, tissues, or organs. Among the different therapeutic approaches considered in Regenerative Medicine, cell-based therapies, namely those using stem cells, are the most explored. In particular, the transplantation of MSCs, which are multipotent cells isolated from mature tissues with paracrine effects and the ability to differentiate into specific lineages (32), has been studied to potentially regenerate damaged tissues or organs (33, 34). This application has been described in several human diseases with clear pro-regenerative effects, such as diabetes mellitus (35), chronic myeloid leukemia (36), cirrhosis (37), pulmonary fibrosis (38), Crohn's disease s (39), heart failure (40), and diseases of the nervous system, such as multiple sclerosis (41), Parkinson's disease (42), and other neurological diseases (43). More recently, new methodologies have begun to be explored to maximize the effectiveness of MSCs as a therapeutic component, namely the use of their secretion products as a substitute for the use of the cells themselves. The set of soluble factors secreted by cells (secretome) includes growth factors, cytokines, chemokines, and glycoproteins, and the vesicular factors include microvesicles and exosomes. As a whole, the pro-regenerative efficacy of these paracrine factors has been shown to be as effective or more effective than the use of cells, without the disadvantages associated with direct cellular administration in a living organism (44).

In addition, there are several transplantation studies using MSCs applied in the dog as a clinical model, namely in spinal cord injury (45), chronic superficial keratitis (46), dermatitis (47), osteoarthritis, and cartilage regeneration (48–50), as well as other musculoskeletal diseases (51).

Transplantation of stem cells in ALS has been described to decrease clinical symptoms, with reported signs of neuroprotection, nervous tissue healing, and increased survival time (52). Other studies showed that intrathecal administration improved motor function, with less neuronal degenerescence (53), and astrogliosis and microgliosis reduction, mainly due to its anti-inflammatory effects (54).

The set of different modalities associated with active exercises (6) and stem cell administration (54) can potentially increase DM dogs' wellbeing in the long term (55).

In regard to neurorehabilitation modalities, functional electrical stimulation (FES) promotes neuromodulation with a low current intensity allowing muscle contraction (56, 57), with short electric pulses that stimulate motor neurons near the motor point or through peripheral afferent stimulation (57, 58). Electrical parameters are usually from 25 to 50 Hz for 15–20 min each session (59).

Additionally, locomotor training based on task repetition intends to achieve neuroplasticity (57), improving muscle mass and oxidative ability (60). This may result in coordinated, consistent (61), and symmetric ambulation (62). Underwater treadmill training may be a fundamental element in the rehabilitation of these dogs, stimulating the neuromuscular system and increasing the range of motion, even when compared to land treadmill training (63, 64).

Therefore, this prospective controlled blinded cohort clinical study aims to evaluate the neural regeneration response ability of DM dogs subjected to an intensive neurorehabilitation protocol with MSCs transplantation. It is hypothesized that the association of this protocol with stem cell transplantation may be a possibility for a positive and longer progression of these dogs.

## 2. Materials and methods

This prospective controlled blinded cohort clinical study was conducted at the Arrábida Animal Rehabilitation Center (CRAA and CR^2^AL, Portugal) between March 2015 and March 2023, after approval from the Lusófona Veterinary Medicine Faculty (Lisbon, Portugal) ethics committee (No. 113-2021) and after signing an informed consent by the owners. Our study has been submitted for publishing on the AVMA Animal Health Studies (AAHSD) website and has been assigned the following number: Study #: AAHSD005642.

### 2.1. Population presentation

The present study included 13 dogs (*n* = 13) with non-ambulatory paraparesis, classified with the open field score (OFS) (65) as OFS 6 or OFS 8, without spinal hyperesthesia and that presented UMN clinical signs compatible with T3-L3 neuro-localization at the neurorehabilitation examination, indicating some degree of chronicity and spasticity in the hindlimbs.

In all dogs, the diagnosis of DM was made by exclusion of immune-mediated myelopathies (e.g., babesiosis and ehrlichiosis); parasitic myelopathies (e.g., leishmaniasis, neosporosis, and toxoplasmosis); and viral myelopathy (e.g., distemper). All were performed by serology of blood and CSF. Cytology of the CSF was also performed to analyze the protein content. CT and/or MRI was performed to exclude T3-L3 compressive myelopathies, such as IVDD Hansen type I and type II; spinal arachnoid cysts; traumatic injury; and vascular injury (fibrocartilaginous embolism).

Followed by the negative results of all aforementioned exams, all dogs had to present a positive genetic test for the SOD1 gene mutation exon 2 (homozygous genotype DM/DM) from the same laboratory (Genevet^®^).

Sample characterization of the population in regard to age, sex, breed, etc., is described in Table 1.

**Table 1:** Population sample characterization (*n* = 13).

	**Total (*n* = 13)**	**Study group (*n* = 8)**	**Control group (*n* = 5)**
Age	≤9 years old: 38.5% (5/13)	≤9 years old: 25% (2/8)	≤9 years old: 60% (3/5)
	>9 years old: 61.5 (8/13)	>9 years old: 75% (6/8)	>9 years old: 40% (2/5)
	Mean: 9.69 years old	Mean: 10.5 years old	Mean: 8.4 years old
Weight	≤30 kg: 61.5% (8/13)	≤30 kg: 75% (6/8)	≤30 kg: 40% (2/5)
	>30 kg: 38.5% (5/13)	>30 kg: 25% (2/8)	>30 kg: 60% (3/5)
	Mean: 30.54 kg	Mean: 29.38 kg	Mean: 32.40 kg
Sex	Male: 46.2% (6/13)	Male: 50% (4/8)	Male: 40% (2/5)
	Female: 53.8% (7/13)	Female: 50% (4/8)	Female: 60% (3/5)
Breed	Pure-breed: 84.6% (11/13)	Pure-breed:75% (6/8)	Pure-breed: 100% (5/5)
	Mixed-breed: 15.4% (2/13)	Mixed-breed:25% (2/8)	
Clinical occurrences	Absent: 61.5% (8/13)	Absent:62.5% (5/8)	Absent: 60% (3/5)
	Present: 38.5% (5/13)	Present: 37.5% (3/8)	Present: 40% (2/5)

### 2.2. Study design

After exclusion, 13 dogs remained in the study regardless of age, weight, sex, breed, and clinical occurrences. All underwent a neurorehabilitation consultation on admission and were randomized by stratification according to the owners' treatment decisions. The study group included dogs whose owners accepted intensive neurorehabilitation with stem cells protocol (INSCP) (*n* = 8) and the control group included dogs subjected to an ambulatory rehabilitation protocol (ARP) (*n* = 5).

Admission consultation and further evaluations throughout the study were recorded (Canon EOS Rebel T6 1300 D camera) and performed by a certified canine rehabilitation practitioner (CCRP) examiner and instructor (Â.M.), who was blinded to the randomization and protocol implemented. The randomization process was completed by a CCRP student (D.G.) who implemented the protocol and worked with the dogs with the remaining rehabilitation team, one other CCRP (A.O.) and one other CCRP student (C.C.). One-third blinded CCRP (A.C.) was responsible for the evaluation of all movies (in regular and slow motion), considering an interobserver disagreement <20%.

The neurorehabilitation consultation took place in a controlled and calm environment, using a 12-cm Halsted mosquito forceps and an 18-cm Taylor hammer, including gait assessment (6-meter walk) and OFS classification; mental state; posture; palpation of the spine; postural reactions; peripheral spinal reflexes (patellar, withdrawal, cranial-tibial, cross-extensor, Babinsky, and cutaneous trunci reflexes); superficial and deep perception of pain; muscle tonus; and flexion/extension range of motion of all joints to assess muscle rigidity and/or spasticity.

Dogs of both groups presented with OFS 6 or OFS 8, clonic patellar and cranial-tibial reflexes, decreased withdrawal reflex in some cases but in others positive for cross-extensor reflex, positive cutaneous trunci reflex until L5, and positive pain perception. All presented hypertonia of the hindlimbs extensor muscle group and hypotonia of the hamstrings muscles, showing also some degree of muscle weakness and atrophy. The representative study algorithm is described in the Supplementary material.

#### 2.2.1. INSCP

The INSCP group included dogs that were under a hospitalization regimen for 2 months and performed the following training (Table 2).

**Table 2:** Description of the protocol for the INSCP group and ARP group.

	**INSCP**		**ARP**	
	**I**	**T**	**I**	**T**
Land treadmill	0.9–2.8 km/h	10–60 min	0.8–1.8 km/h	15–30 min
	Starting 5 times/day until 2 times/day on last week		2 times/day 4–5 days/week	
Underwater Treadmill	**I**	**T**	**I**	**T**
	1–3.5 km/h	5 min−1 h	0.8–1.8 km/h	10–40 min
	1 time/day 5 days/week		1 time/day 4–5 days/week	
Kinesiotherapy	Up/down stairs and ramps Walking on different floor surfaces Cavaletti rails		–	
	2–5 times/day 4–5 days/week 5–10 min			
Electrical stimulation	FES			
	40–60 Hz 10–46 mA Pulse duration 1:4 Ramp up 4 s Plateau 8 s Ramp down 2 s			
	2–3 times/day 5 days/week		1 time/day 4–5 days/week	

I, intensity; T, time; FES, functional electrical stimulation; Hz, hertz; mA, milliamperes; min, minutes; km/h, kilometers per hour.

##### 2.2.1.1. Locomotor training

The locomotor training implemented was based on fast step-cycle repetitions, always applied in a quiet environment, and ideally with musical stimulation (66, 67). Due to the presentation of clonic reflexes, bicycle movements were only necessary to modulate the rhythm of the step cycle, rarely requiring tail and perineal stimulation.

In these cyclic movements, stretching of the hindlimbs should be avoided, but with vigorous cutaneous afferent receptors stimulation on the treadmill surface (68). Early implementation of the quadrupedal training is intended for complete stimulation.

**Figure 1:**
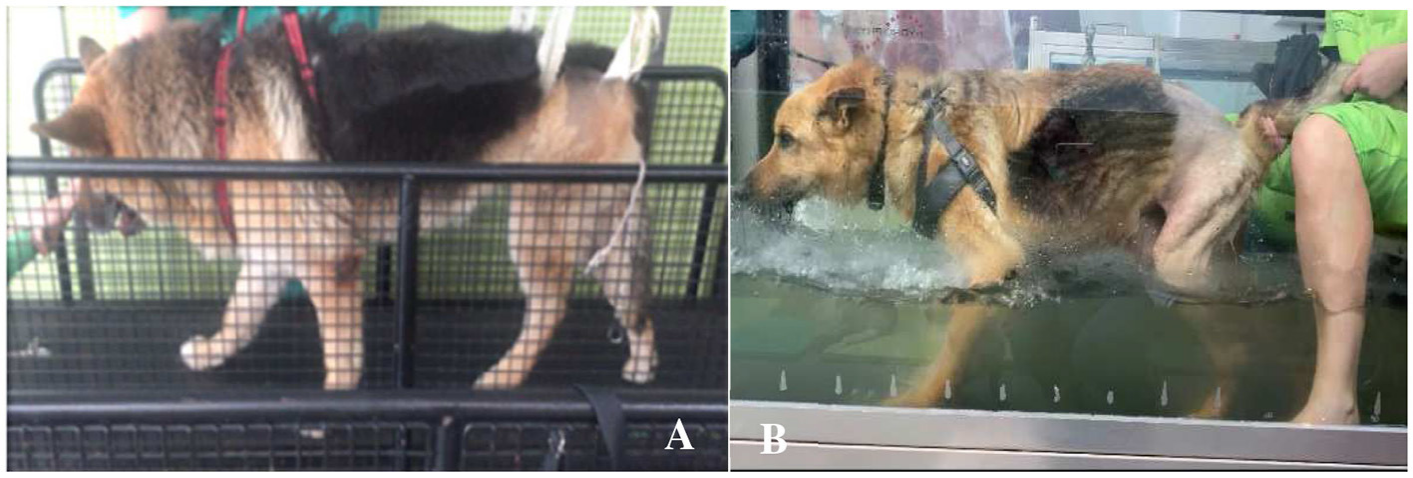
Locomotor training. **(A)** Land treadmill training. **(B)** Underwater treadmill training.

Land treadmill training was initiated with 0.9 km/h aiming to achieve 2.8 km/h for 10 min until a maximum of 60 min, initially with 5 repetitions a day in the first week, until 2 repetitions in the last week (Figure 1A). Underwater treadmill training was initiated at admission or the following day, always with water temperature nearly 26°C, beginning with 5 min until reaching 1 h, 5 days/week, and speeds of 1 km/h until 3.5 km/h (Figure 1B), with care for signs of overtraining (67) (Table 2).

Kinesiotherapy exercises were also included in the protocol, such as walking up/down stairs and ramps; on different floor surfaces; and cavaletti rails. These circuits were done 2–5 times/day, 4–5 days/week for 5–10 min.

##### 2.2.1.2. Electrical stimulation

Functional electrical stimulation consisted of nerve stimulation with one electrode applied on L7-S1 anatomical region and the other electrode on the hamstring's muscle motor point. Parameters were 40–60 Hz, 10–36 mA, pulse duration 1:4, ramp up 4 s, plateau 8 s, and ramp down 2 s. These were done 2–3 times/day, 5 days/week, according to neurological evolution.

##### 2.2.1.3. Mesenchymal stem cells preparation

The preparation of the cell-based therapies used in this clinical study followed an adaptation of the protocol patented by the University of Porto (WO2019175773—Compositions for use in the treatment of musculoskeletal conditions and methods for producing the same leveraging the synergistic activity of two different types of mesenchymal stromal/stem cells (Regenera^®^), PCT/IB2019/052006) (69). Previously harvested, isolated, and characterized (data not yet published) canine synovial membrane MSCs (cSM-MSCs) were used in this study. All SM-MSCs used in this study were obtained from the synovial membrane of a healthy and young donor dog and collected using arthroscopy. Before the harvest, the owners signed an informed consent, and the animal underwent a complete medical and orthopedic evaluation, as well as a screening for infectious and contagious diseases. After harvesting the synovial membrane, cell isolation was performed as previously described (70). The applied therapeutic combination consisted of the administration of allogeneic cSM-MSCs suspended in autologous serum. Total blood was previously collected from the animals to be treated, in dry blood collection tubes. After coagulation, the tubes were centrifuged at 2,300 rpm for 10 min and their supernatant (serum) was isolated and collected. Then, the serum was inactivated by immersion in a water bath at 56°C for 20 min followed by cooling on ice. Finally, the serum was centrifuged and filtered using a 0.22-μm syringe filter and stored at −20°C until further use. For each administration, ~5 × 10^6^ cryopreserved low passage (P2) allogeneic cSM-MSCs were thawed in a 37°C water bath. After thawing, the cell content was transferred to a sterile tube and diluted in sterile and previously filtered DPBS. The mixture was centrifuged at 1,600 rpm for 10 min. After centrifugation, the supernatant was eliminated with a single movement, and the cell pellet was resuspended in ~2 ml of autologous serum previously thawed in a 37°C water bath. Cell counting and viability were determined through the Trypan Blue exclusion dye assay (Invitrogen^TM^) using an automatic counter (Countess II FL Automated Cell Counter, Thermo Fisher Scientific^®^). The cell number was then adjusted to 5 × 106 cells/ml. Then, 2 ml of the solution of cSM-MSCs suspended in autologous serum was transferred to a perforable capped vial and preserved on ice until the time of administration.

##### 2.2.1.4. Mesenchymal stem cells transplantation

The stem cells transplantation was performed on day 30 (T1) by the head of the neurology department of the Veterinary Medicine Faculty—Lisbon University (A.F.) and his team, as follows: vascular access and fluid therapy during the procedure; induction with propofol (2 mg/kg); endotracheal intubation and anesthetic maintenance with isoflurane; trichotomy of the dorsal cervical region and asepsis; intrathecal transplantation based on the landmarks of the occipital prominence and the spinous process of C2, with a 22-gauge pencil-point needle inserted cranially to the atlas wings edge, penetrating the subarachnoid space; extraction of 1 ml of CSF for a sterile tube; administration of 1 ml of stem cells; and vertical positioning of the dog for 10 min.

#### 2.2.2. Ambulatory rehabilitation protocol

The ambulatory rehabilitation protocol group was under an ambulatory regimen, 4–5 times a week. This protocol was based on locomotor training and electrical stimulation with few alterations (Table 2). Moreover, there was no MSCs administration in these dogs.

##### 2.2.2.1. Locomotor training

The main difference in locomotor training was the frequency. Dogs of the ARP group only performed two repetitions for 15–30 min of land treadmill training for the session. Regarding the underwater treadmill, it was performed once for 10–40 min. Both training had speeds of 0.8–1.8 km/h, according to each dog's tolerance, and sessions were done 4–5 days/week.

##### 2.2.2.2. Electrical stimulation

As for the functional electrical stimulation, the protocol was the same for both groups; however, the ARP group only had one repetition each session, 4–5 days/week.

### 2.3. Outcomes and monitorization

Dogs of both groups were admitted (T0) and evaluated according to their neurological status and classified with the OFS. The INSCP dogs were hospitalized in the rehabilitation center and after the first month of intensive rehabilitation (T1), they were subjected to stem cell transplantation at day 30 (T1), followed by the second month of rehabilitation (T2) and medical release at the end. The ARP dogs were under ambulatory sessions of rehabilitation during the same 2 months.

**Figure 2:**
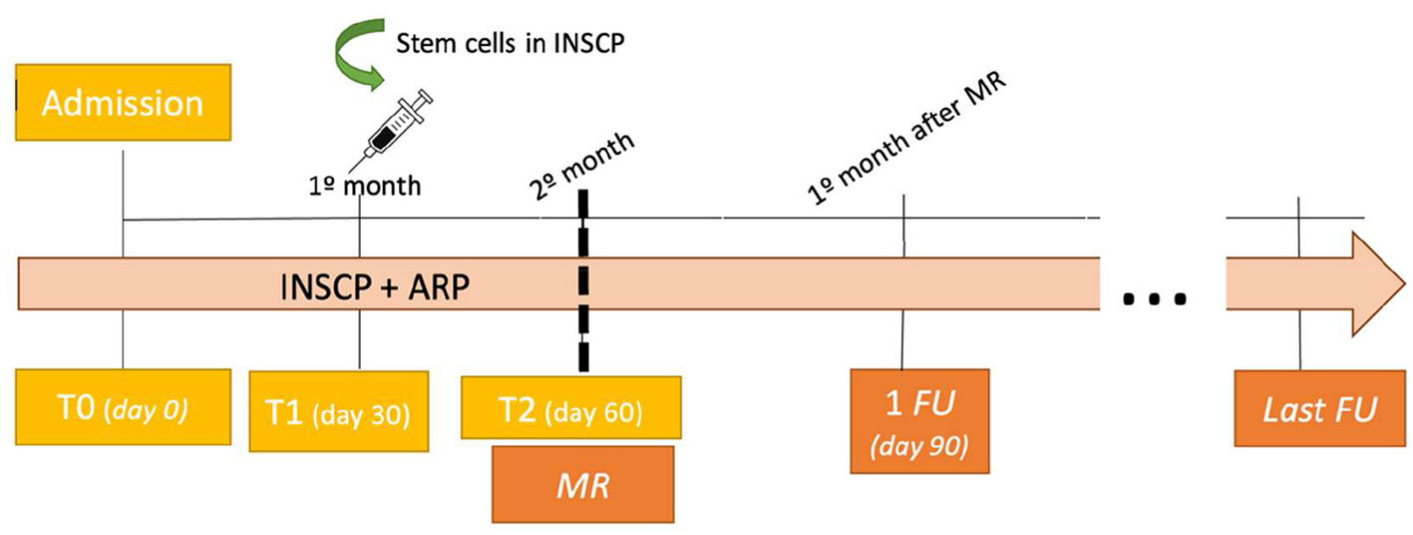
Outcomes and monitorization algorithm (*n* = 13). INSCP, intensive neurorehabilitation stem cell protocol; ARP, ambulatory rehabilitation protocol; MR, medical release; FU, follow-up.

Dogs of both groups were evaluated at the end of day 30 (T1) and day 60 (T2). Follow-ups were performed and recorded 1 month after medical release (1FU) and the last follow-up was according to each dog, as presented in Figure 2.

### 2.4. Statistical analysis

Records were documented using Microsoft Office Excel 365^®^ (Microsoft Corporation, Redmond, WA, USA) and processed in IBM SPSS Statistics 25^®^ (International Business Machines Corporation, Armonk, NY, USA) software. Shapiro–Wilk normality test, arithmetic means, minimum, maximum, standard deviation (SD), and standard error of the mean (SEM) were recorded for continuous variables age and weight. Descriptive statistics with frequency analysis was performed for all categorical nominal variables. Chi-square tests were also performed to verify relevant analogies proven by a *p*-value of <0.05. One sample *t*-test was used for comparison with previously published studies. The estimated marginal means for comparison at each time point regarding the OFS scores and survival time were performed using Analysis of Variance (One-Way ANOVA) for repeated measures and the post-Tukey HSD test. In addition, the Kaplan–Meier survival analysis was performed.

## 3. Results

In this prospective controlled blinded cohort clinical study, from the 13 homozygotic DM dogs, the binominal qualitative variable sex presented 53.8% (7/13) of female and 46.2% (6/13) of male dogs. For the binominal variable breed, it was observed that 15.4% (2/13) were mixed-breed dogs and 84.6% (11/13) were pure-breed dogs, including German Shepherds (*n* = 7) as the most prevalent, followed by the Collie (*n* = 1), Alaskan Malamute (*n* = 1), Weimaraner (*n* = 1), and Setter (*n* = 1).

Descriptive analysis of the continuous quantitative variables for age and weight is reported in the Supplementary material, with a mean of 9.69 years and 30.54 kg, and a verified normal distribution by the Shapiro–Wilk Normality Test (*n* < 50) for age (*p* = 0.684) and weight (*p* = 0.233).

Clinical occurrences were absent in 61.5% (8/13) and present in 38.5% (5/13), of which three belonged to the study group and two to the control group.

**Figure 3:**
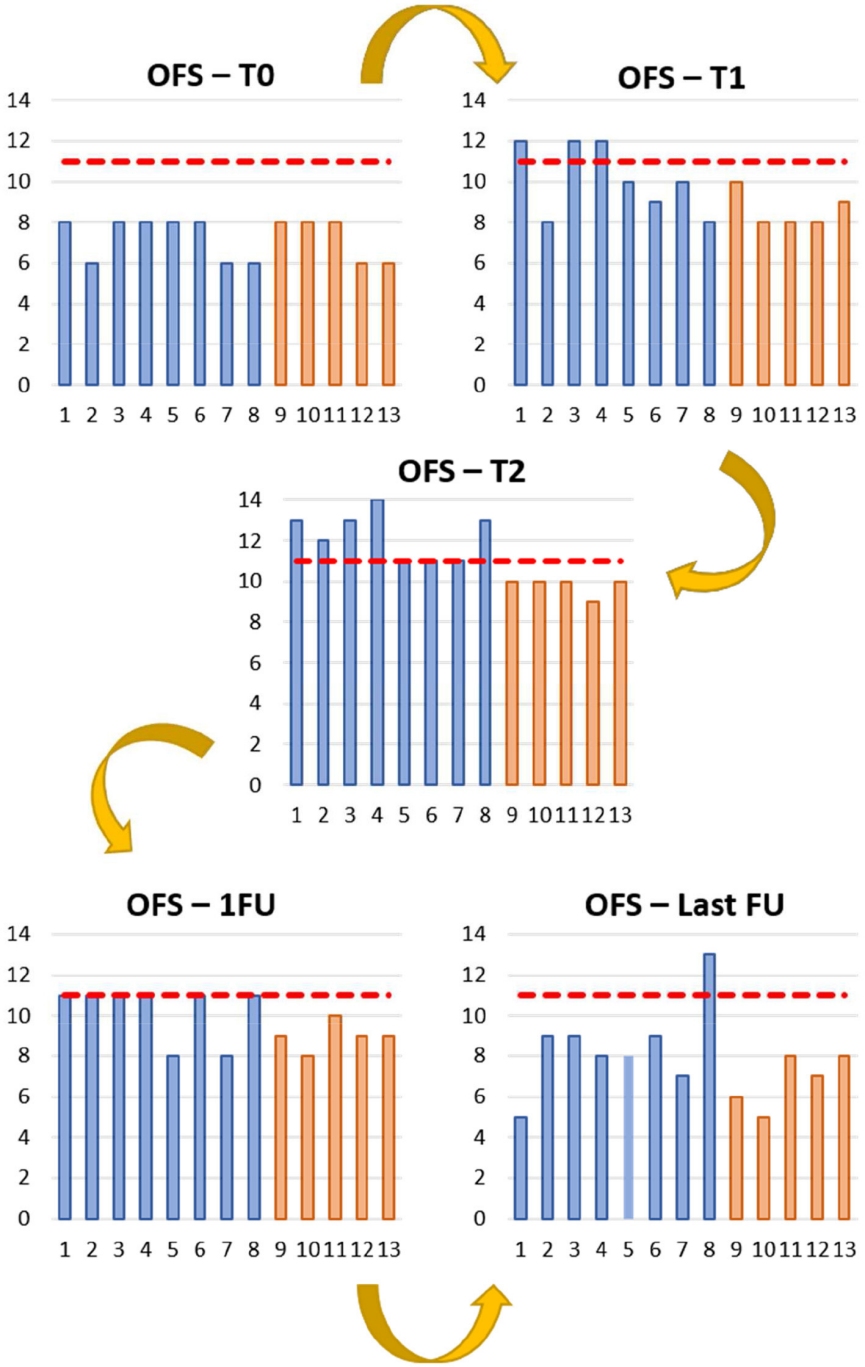
Open field score (OFS) reports from the study (blue) and control (orange) Groups during the different time points. T0 (day 0); T1 (day 30); T2 (day 60); 1FU (day 90); Last FU (last follow-up). Y-axis: OFS; X-axis: Dogs 1 to 13; — OFS 11.

The OFS outcomes were documented for both INSCP and ARP groups, at each time point (T0, T1, T2, 1FU, and Last FU), and ambulation was considered when OFS ≥ 11, as shown in Figure 3. Regarding the OFS classification, inter-observer disagreement was 11%.

**Figure 4:**
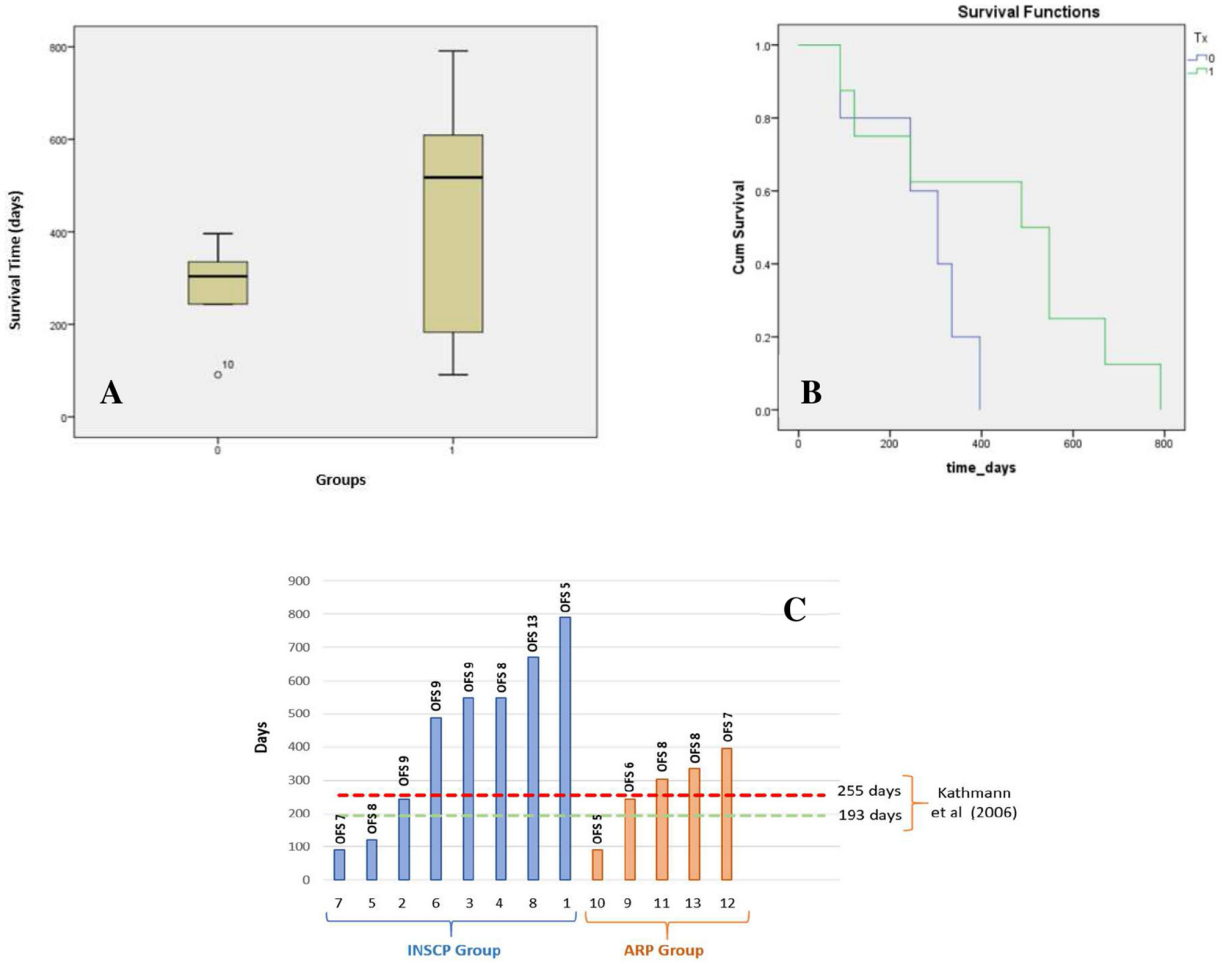
Study's population survival time. **(A)** Survival time (days) boxplot for INSCP (1) and ARP (0) groups; X-axis: groups (INSCP and ARP); Y-axis: survival time (days). **(B)** Kaplan–Meier survival analysis; y-axis: cumulative (Cum) survival; X-axis: survival time (days) from INSCP group (green) and ARP group (blue). **(C)** Survival time of the INSCP and ARP groups with open field score (OFS) for each dog on the last follow-up and comparison with Kathmann et al. (6); Y-axis: days; X-axis: dogs from INSCP group (blue) and ARP group (orange).

In the present study, a total mean survival time of 375 days was obtained, with the INSCP group presenting a mean survival time of 438 days compared to 274 days for the ARP group, as shown in Table 3 and Figure 4A. The minimum days of survival time for each group was 91 days, although the INSCP group presented a maximum of 791 days compared with 396 days for the ARP group. Comparing both groups according to survival time, differences were also demonstrated by the survival analysis (Kaplan–Meier), as shown in Figure 4B.

**Table 3:** Descriptive analysis of survival time (days).

		**Total (*n* = 13)**	**Study group (*n* = 8)**	**Control group (*n* = 5)**
Survival time (days)	Mean	375	438	274
	Median	335	517.5	304
	Mode	91	548	91
	Variance	49,933.897	66,133.411	13,473.5
	SD	223.459	257.164	116.075
	Minimum	91	91	91
	Maximum	791	791	396
	SEM	61.976	90.921	51.91

SD, standard deviation; SEM, standard error of mean.

By the Chi-square tests, age [$X_{\left(1,\text{n}\;=\;13\right)}^{2}$ = 0.008, *p* = 0.928], weight [$X_{\left(1,\text{n}\;=\;13\right)}^{2}$ = 1.593, *p* = 0.207], and sex [$X_{\left(1,\text{n}\;=\;13\right)}^{2}$ = 0.124, *p* = 0.725] did not show any interference in the survival time.

Kathmann et al. (6) demonstrated a mean survival time of 255 days for nine dogs and 130 days for six dogs, considering intensive and moderate physiotherapy, respectively. The present study reported survival time (days) for each dog of both groups and the OFS results at the last follow-up. This comparison is presented in Figure 4C.

When comparing both intensive and moderate physiotherapy of Kathmann et al. (6) (mean survival time 193 days) with the present study (*n* = 13), significance in the one sample *t*-test [*t*_(12)_ = 2.932, *p* = 0.013] was shown. Although, when considering the INSCP group (*n* = 8) with the intensive physiotherapy group (*n* = 9) from Kathmann et al. (6), with a mean survival time of 255 days, no significance was observed [*t*_(7)_ = 2.009, *p* = 0.085].

**Figure 5:**
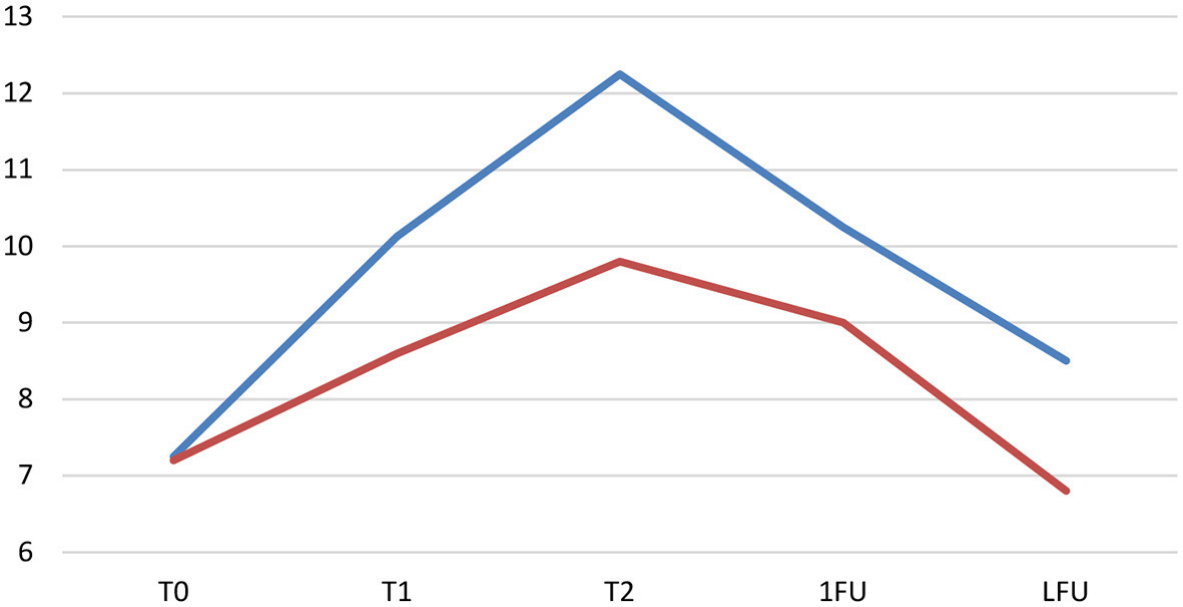
Evolution of the open field score (OFS)—estimated marginal means in both the INSCP group (blue) and ARP group (orange). Y-axis: OFS; X-axis: T0 (day 0); T1 (day30); T2(day 60); 1FU (day 90); LFU, last follow-up.

Considering the OFS of the INSCP group and ARP group, a significance [*F*_(1,64)_ = 7.490, *p* = 0.008] between them by the One-Way ANOVA was detected, as well as a significance [*t*_(63)_ = −2.737, *p* = 0.008] by the independent sample *t*-test. That finding could also be verified in the OFS estimated marginal means evolution chart (Figure 5).

For the INSCP group, regarding the OFS evolution for each time point, a significant difference [*F*_(4,64)_ = 14.287, *p* ≤ 0.001] was reported. The post-Tukey HSD test was performed between time points and showed that from T0 until the first follow-up, significances were always demonstrated (*p* = 0.003 with T1; *p* ≤ 0.001 with T2 and *p* = 0.001 with 1FU). Considering T1 until T2 (1 month after stem cell transplantation), results showed significance (*p* = 0.039), and, also, from T2 until the last follow-up of each dog (*p* ≤ 0.001), a consistent descending curve was observed, as shown in Figure 5.

## 4. Discussion

Distribution in regard to sex was higher for female dogs with 53.8% (7/13), agreeing with Coates et al. (1) who reported a total of 21 dogs with five male and 16 female dogs. Also, a similar prevalence was seen in a study by March et al. (15) with 18 dogs, four male and 14 female dogs. However, many authors referred to the absence of sexual predisposition in DM (3, 8).

As for breed predisposition, which is already stated in the literature (3), the German shepherd had the higher prevalence with 53.8% (7/13), which agrees with most research on DM (9, 12, 71–73). Although the histological diagnosis is described as a mere 0.19%, this percentage increases drastically to 2.01% in the German shepherd (1). In 2018, Donner et al. (74) studied the frequency and distribution of genetic diseases in dogs of mixed and pure breeds, pointing to DM as the number one of the rankings. However, it cannot be ignored the complex etiology of this disease, such as the immunologic (75), metabolic, nutritional (76, 77), oxidative stress (1), and excitotoxic effects (78).

The average age of onset of clinical signs was 9.69 years old, similar to those reported in the literature (3, 7). In contrast, mean weight had high variability between studies according to the most represented breeds. For example, a study in Pembroke Welsh Corgi reported an average of 11 kg (1, 17), much lower than the 30.54 kg of this study. Other studies with larger breeds presented mean weights >25 kg, such as Polizopolou et al. (31) and Johnston et al. (72).

The 13 dogs that were randomized for the INSCP group and ARP group according to the owner's decision of treatment presented a normal distribution by the Shapiro–Wilk Normality Test for age (*p* = 0.684) and weight (*p* = 0.233) and were compared in the study. Of the total of these dogs, and in both groups, nearly 60% did not present any type of clinical occurrences, probably due to the inclusion criteria (non-ambulatory paraparesis OFS 6 or 8).

Over the evaluation times (T0, T1, and T2) and first follow-up, an increase of OFS ≥11 was observed in most dogs of the INSCP group, indicating ambulation (79) and reported for each time point with significance (*p* ≤ 0.001). This was also supported by the post-Hukey test, with significances always present between T0 and 1FU (*p* = 0.003 with T1; *p* ≤ 0.001 with T2 and *p* = 0.001 with 1FU). Although, all dogs in both groups appeared to have an improvement regarding their sensorimotor status up to the first follow-up (1FU).

This increase in neurological status between T0 and T1 was mainly due to the association between locomotor training with electrical stimulation modalities, as expected and already mentioned by Kathmann et al. (80) and Miller et al. (81). These are neurorehabilitation concepts (82), which allow neural control of movement after changing sensory inputs (83) by the work of intrinsic ability to generate rhythmic movements (84).

The locomotor training helps in relearning stepping with a coordinated and modulated ambulation pattern (57, 67) based on a dynamic interaction between afferent inputs for all functional receptors (e.g., proprioceptive and biomechanical) (85, 86). This can be applied through bodyweight-supported treadmill training (BWSTT), which helps reduce spasticity by replacing abnormal hyperexcitable sensory firing with functional signaling, decreasing muscle spasm/contraction (87, 88), and improving coordination and ambulation (89), essential for affected DM dogs with UMN signs, for example, clonic reflexes.

One of the locomotor training strategies resides in the stimulation of descending propriospinal neurons essential to activate neural networks leading to stepping recovery (90, 91).

Furthermore, FES stimulates the unnatural recruitment of muscle fibers by engaging large-diameter motor neurons that have fast conduction velocity fibers, instead of recruiting small-diameter motor neurons, which are slower and fatigue-resistant (92). This modality has been reported to modulate neural circuitry (93, 94) and allows repetitive cycles of inputs/outputs, suppressing excessive afferent synaptogenesis and improving remaining neural circuits (95).

DM dogs present changes in myofibers and connective tissues that can alter the electrical properties, with progressive decrease in myofiber size, and increase in fat and connective tissue that may increase resistance (96). In the early stage of DM, dogs are characterized by disuse atrophy (3, 97), but in late DM, lower motor neuron signs begin, such as neurogenic atrophy. This is always with a caudodistal location associated with atrophy secondary to distal axonopathy (25, 96), with myelin profiles replaced by large areas of astrogliosis (9, 13, 72, 98).

In Figures 3, 5, the higher OFS results were from T1 to T2 mainly in the INSCP group, with a marked significance between groups (*p* = 0.039). Thus, after intrathecal transplantation (IT) of stem cells, which results in indirect cell distribution through the central nervous system (CNS) by the CSF (99). This technique intends to avoid the “first-pass” pulmonary effect and cell entrapment, limiting their therapeutic effect (100, 101). In addition, IT avoids the blood-brain barrier, allowing to focus the cells' trophic effects directly on the CNS (99, 102, 103).

The MSCs applied were synovial membrane-derived mesenchymal cells. MSCs can promote cell survival and tissue repair by increasing paracrine secretion of neurotrophic and angiogenic factors (104–106), such as brain-derived neurotrophic factor (BDNF), ciliary neurotrophic factor (CNTF) (107, 108), neurotrophin-1 (NT-1), neurotrophin-3 (NT-3), nerve growth factor (NGF), fibroblast growth factor (FGF), and glial cell-derived neurotrophic factor (GDNF) (109–114). All these support neurogenesis, axonal growth, re-myelinization, and cell metabolism (114–119), contributing also to the ability to recruit oligodendrocytes precursors (41). Recently, the therapeutic effects and also the immunomodulatory potential of SM-MSCs were demonstrated after application in dogs in Veterinary Medicine and after a preliminary characterization of its secretome (70).

In this prospective controlled blinded cohort clinical study, the mean survival time was 375 days in the total population, with the INSCP group having 438 days compared to the ARP group with 274 days. In the INSCP group, there was a maximum survival of 791 days (Table 3), which can be observed in the survival time boxplot (Figure 4A) and in the Kaplan–Meier survival analysis (Figure 4B).

Thus, the main difference may be linked to the MSCs transplantation in the INSCP group, in addition to intensive neurorehabilitation. MSCs potentiate aquaporin 1 and CXCR4 expression, two membrane proteins involved in cell migration, which help in the “homing” mechanisms (114), potentiated by the vertical position of the dog for 10 min that was included after this stem cells transplantation technique.

As previously explained and considering the study design, the selection of both groups was according to the owners' treatment decision. Although there was no clear evidence of the sole role of the MSCs given the differences of both protocols and even if the intensive neurorehabilitation demonstrated to have always a slight improvement compared to the ARP, an evident positive progression was observed after transplantation (T1), with all dogs from the INSCP achieving ambulation in this period (Figure 3). It is the authors' opinion that these results were not only due to the same intensive training applied until discharge, suggesting that it was also the combination of the neurorehabilitation intensive protocol with the MSCs transplantation that allowed this evolution.

DM implies Wallerian degeneration that causes fragmentation of damaged axons, generating debris and extracellular deposition of myelin-related molecules with chondroitin sulfate proteoglycans inhibiting neural regeneration and neuroplasticity in the long term. To delay this degenerative progression of the disease, the role of GDNF is reported (120), possibly contributing to the increase of dendrites length, number of dendrites, or a combination of both. Thus, aiming to achieve long-term potentiation due to reactivable remaining tissue (95).

Comparison of the total population (*n* = 13) with previous literature (6) considering dogs submitted to moderate or intensive physiotherapy (*n* = 15), by the one-sample *t*-test, revealed a significant difference (*p* = 0.013), as can be seen in Figure 4C with only three dogs (two from the INSCP and one from the ARP) achieving a survival rate lower than the 193 days reported by Kathmann et al. (6).

The mean survival time is compared between the mentioned study's intensive physiotherapy group (255 days) and our INSCP group (438 days). Although an increase of nearly 70% was visible, there was no significance obtained. Therefore, the authors believe that intensive rehabilitation is the minimum necessary that these dogs need to increase their survival time as it has already been proven, but stem cell transplantation may delay the progression and increase the survival time. However, there is not enough data, and research must continue.

When comparing the OFS results between the INSCP and ARP groups (one-way ANOVA and independent-samples *t*-test), there was also a clear significant difference. Figure 5 demonstrates this disparity in relation to the estimated marginal means at each moment of the study. In the same figure, there is a significant decrease from medical release to the last follow-up, justified by the degenerative and progressive nature of the disease, where glial cells play a critical role in the pathogenesis of this type of neurodegenerative diseases, such as ALS (121–123). Few studies also report the importance of increased glial cells and inflammatory molecules, partially responsible for the disease progression (15, 124, 125).

In the near future, it would be of main interest to have a quick and feasible test to assess the phosphorylated neurofilament heavy subunit (pNF-H) protein, a major structural component of large myelinated axons, which could be useful to measure axonal damage (23, 126–128).

The major cause of death was euthanasia due to the progression of DM with 69.2% (9/13). Euthanasia was perfromed after the last follow-up of each dog only if they presented the following clinical signs: thoracolumbar weakness; hindlimbs muscle mass atrophy; decreased hindlimbs spinal reflexes and cutaneous trunci reflex; decreased abdominal muscle tone; and decreased trunci balance.

The remaining 30.8% (4/13) died due to internal medical causes, all belonging to the INSCP group. From these, three dogs had cardiorenal syndrome secondary to dilated cardiomyopathy (with a survival time of 244, 487, and 670 days) and one dog was secondary to splenic neoplasia (with a survival time of 781 days). The one dog that died from dilated cardiomyopathy had a positive histopathologic exam for degenerative myelopathy (Necropsy Report N°40/20).

For neurorehabilitation in DM dogs, it is important to the notion of astrogliosis, which happens mainly in the dorsal horn region, that it gives rise to the dorsal spinocerebellar tracts (72). In this disease, the lesion will focus on the dorsal funiculus, including ascending and descending tracts within the dorsal portion of the lateral funiculus and the ascending pathways within the dorsal funiculus (3, 25). These may explain the constant loss of proprioception, leading to the need for locomotor training and association with kinesiotherapy exercises (e.g., alternating water levels, backward treadmill, and home exercises). In this study after T2, all dogs were only stimulated by home exercises 3 times/day, such as leach walking (15 min), cavaletti rails (2 min), stairs (2 min), ramps (2 min), and proprioceptive unbalance exercises (5 min).

Therefore, these results are in agreement with previous literature and suggest that the combination of AD-MSCs transplantation with intensive neurorehabilitation protocols may provide a functional improvement (129) with no long-term related side effects (130, 131). The same association was already applied in ALS patients (120, 131), resulting in a safe and feasible approach (132, 133).

It is possible to conclude that this INCSP may be an option for a long progression of DM dogs with quality of life and without suffering. In the author's opinion, its implementation should be as early as possible and not in the late stage of DM.

The limitations of this study were the small sample size and the randomization process based on owner consent. In addition, there was only one case with a definitive histological diagnosis, lack of biomarkers, and the absence of intensive neurorehabilitation after medical release.

## Data availability statement

The raw data supporting the conclusions of this article will be made available by the authors, without undue reservation.

## Ethics statement

The animal study was reviewed and approved by CEBEA-Comissão de Ética e Bem-estar Animal da Universidade Lusófona (Lusófona Veterinary Medicine Faculty, Lisbon, Portugal, No. 113-2021). Written informed consent was obtained from the tutors for the participation of their animals in this study.

## Author contributions

All authors listed have made a substantial, direct, and intellectual contribution to the work and approved it for publication.
