# Supplementary material for: Intensive neurorehabilitation and allogeneic stem cells transplantation in canine degenerative myelopathy
Source: Front Vet Sci. 2023 Jul 13;10:1192744. doi: 10.3389/fvets.2023.1192744 (PMC10374290; doi:10.3389/fvets.2023.1192744)
Supplement: Supplementary file 1 [file Data_Sheet_1.PDF]

Supplementary Material

Intensive Neurorehabilitation and allogeneic stem cells transplantation in canine degenerative myelopathy

Débora Gouveia[1,2,3], Jéssica Correia[1,3], Ana Cardoso[1,2], Carla Carvalho[1], Ana Catarina Oliveira[1,2], António Almeida[4], Óscar Gamboa [4], Lénio Ribeiro [3], Mariana Branquinho[5,6,7], Ana Sousa[5,6,7], Bruna Lopes[5,6,7], Patrícia Sousa[5,6,7], Alícia Moreira[5,6,7], André Coelho[5,6,7], Alexandra Rêma[5,6,7], Rui Alvites[5,6,7,8], António Ferreira[4,7,9], Ana Colette Maurício[5,6,7*], Ângela Martins[1,2,3]

[1] Arrábida Veterinary Hospital, Arrábida Animal Rehabilitation Center, 2925-538 Setubal, Portugal

[2] Superior School of Health, Protection and Animal Welfare, Polytechnic Institute of Lusophony, Campo Grande, 1950-396 Lisboa, Portugal

[3] Faculty of Veterinary Medicine, Lusófona University, Campo Grande, 1749-024 Lisboa, Portugal

[4] Faculty of Veterinary Medicine, University of Lisbon, 1300-477 Lisboa, Portugal

[5] Departamento de Clínicas Veterinárias, Instituto de Ciências Biomédicas de Abel Salazar (ICBAS), Universidade do Porto (UP), Rua de Jorge Viterbo Ferreira, nº 228, 4050-313 Porto, Portugal

[6] Centro de Estudos de Ciência Animal (CECA), Instituto de Ciências, Tecnologias e Agroambiente da Universidade do Porto (ICETA), Rua D. Manuel II, Apartado 55142, 4051-401, Porto, Portugal.

[7] Associate Laboratory for Animal and Veterinary Science (AL4AnimalS), Lisboa, Portugal.

[8] Instituto Universitário de Ciências da Saúde (CESPU), Avenida Central de Gandra 1317, 24 4585-116 Gandra PRD, Portugal.

[9] CIISA - Centro Interdisciplinar-Investigação em Saúde Animal, Faculdade de Medicina Veterinária, Av. Universi dade Técnica de Lisboa, 1300-477 Lisboa, Portugal

*** Correspondence:**
acmauricio@icbas.up.pt; ana.colette@hotmail.com

1 Supplementary Figures and Tables

1.1 Supplementary Figures

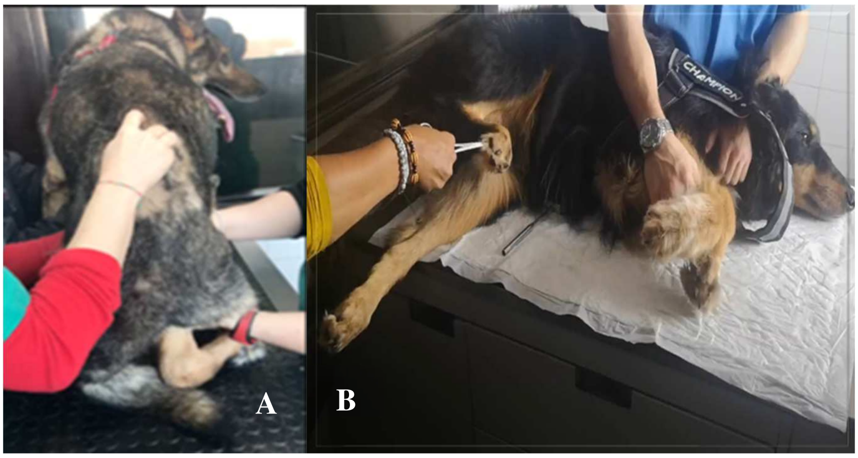

Supplementary Figure 1. Neurorehabilitation examination. A) Palpation of the spine. B) A dog presenting the cross-extensor reflex.

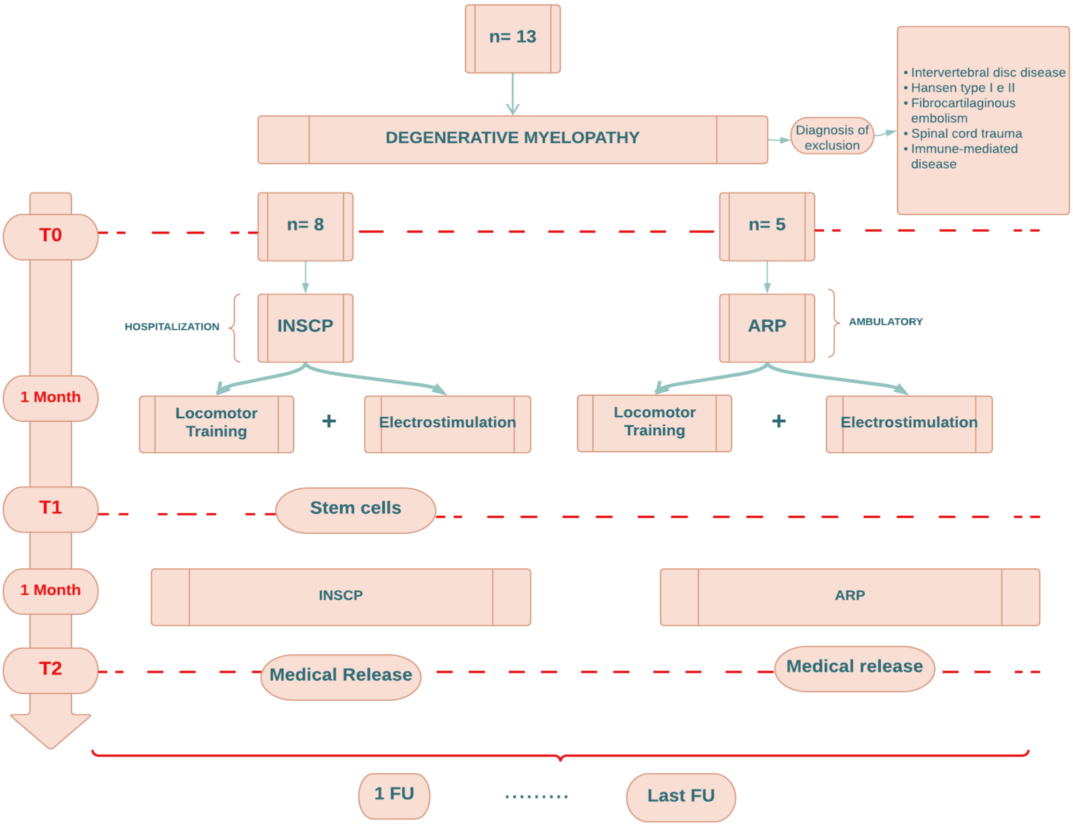

Supplementary Figure 2. Clinical study representative algorithm (n=13). INSCP: Intensive neurorehabilitation stem cell protocol; ARP: Ambulatory rehabilitation protocol; T0: admission day; T1: day 30; T2: day 60; FU: Follow-up.

1.2 Supplementary Tables

Supplementary table 1. Locomotor training protocol

	Land treadmill	Underwater treadmill
**1st week**	**Speed:** 0.9-1.2 km/h<br>**Duration:** 10-20 min<br>**Frequency:** 5 times/day,<br>6 days/week	**Speed:** 0.9-1.2 km/h<br>**Duration:** 10 min<br>**Frequency:** once/day, 5 days/week
**2nd week**	**Speed:** 1.2-2 km/h<br>**Duration:** 20-30 min<br>**Frequency:** 4 times/day,<br>6 days/week	**Speed:** 1.2-2 km/h<br>**Duration:** 20 min<br>**Frequency:** once/day,<br>5 days/week
**3rd week**	**Speed:** 2-2.8 km/h<br>**Duration:** 30-40 min<br>**Frequency:** 3 times/day,<br>6 days/week	**Speed:** 2-2.8 km/h<br>**Duration:** 30 min<br>**Frequency:** once/day,<br>5 days/week
**4th week**	**Speed:** 2.8-3.2 km/h<br>**Duration:** 40-60 min<br>**Frequency:** 2 times/day,<br>6 days/week	**Speed:** 2.8-3.2 km/h<br>**Duration:** 60 min<br>**Frequency:** once/day,<br>6 days/week

Supplementary table 2. Kinesiotherapy protocol

		Kinesiotherapy
INSCP	1st week	Walking in different surfaces<br>5 minutes; 3 times/day.
	2nd week	Walking in different surfaces; 5 minutes; 3 times/day<br>*Cavaletti rail;* 5 minutes; 2 times/day
	3rd week	Walking in different surfaces; 10 minutes; 2 times/day<br>*Cavaletti rail;*10 minutes; 2 times/day
	4th week	Walking in different surfaces;10 minutes; 1 time/day<br>*Cavaletti rail;* 10 minutes; 1 time/day<br>Up and down stairs/ramps; 5 minutes; 3 times/day

Legend: INSCP - Intensive neurorehabilitation with stem cells protocol

Supplementary table 3. Electrical stimulation protocol

		Week	Hz	mA	Times/day
**Electrical Stimulation**	**FES**	1st			4
		2nd			3
			40-60	10-36	
		3rd			2
		4th			1

Legend: FES – Functional electrical stimulation; Hz- Hertz; mA: milliamperes

Supplementary table 4. Ambulatory rehabilitation protocol

ARP									
**Land Treadmill**			**Under Water Treadmill**		**Kinesiotherapy**	**Electrical Stimulation (FES)**			
**I**	**F**	**T**	**I**	**T**		**Hz**	**mA**	**F**	
0,8-1,8	2	15-30	0,8-1,8	10-40	Walking in different surfaces; 10minutes; 2 times/day<br>*Cavaletti rail*; 5minutes; 2 times/day	40-60	10-36	1 (4-5 times/week)	

Legend: ARP – Ambulatory Rehabilitation Protocol; FES – Functional Electrical Stimulation; I – Intensity (km/h) ; F – frequency; T – time (minutes). Hz- Hertz; mA: milliamperes

Supplementary table 5. Descriptive analysis for age and weight

		Total (n=13)
**Age**	Mean	9.69
	Median	10
	Mode	10
	Variance	4.564
	SD	2.136
	Minimum	5
	Maximum	13
	SEM	0.593
	Shapiro-Wilk Normality Test	0.684
**Weight**	Mean	30.54
	Median	29
	Mode	27
	Variance	35.936
	SD	5.995
	Minimum	22
	Maximum	45
	SEM	1.663

	Shapiro-Wilk Normality Test	0.233

Legend: SD - standard deviation; SEM - Standard Error of Mean
